# Investigation of SARS-CoV-2 in tear and conjunctival secretions of hospitalized patients with clinically-confirmed COVID-19 pneumonia

**DOI:** 10.1186/s12879-021-06630-6

**Published:** 2021-09-06

**Authors:** Yunus Karabela, Semsi Nur Karabela, Mehmet Ozbas, Havva Kasikci, Kadriye Kart Yasar

**Affiliations:** 1 Opticianry Program, University of Health Sciences, Uskudar, Istanbul, Turkey; 2 Department of Infectious Diseases and Clinical Microbiology, Bakirkoy Dr. Sadi Konuk Training and Research Hospital, University of Health Sciences, Istanbul, Turkey; 3 Department of Ophthalmology, Bakirkoy Dr. Sadi Konuk Training and Research Hospital, Bakirkoy, Istanbul, Turkey; 4 Molecular Microbiology Laboratory, Bakirkoy Dr. Sadi Konuk Training and Research Hospital, Bakirkoy, Istanbul, Turkey; 5 Opticianry Program, Hamidiye Vocational School of Health Services, University of Health Sciences, Mekteb-i Tibbiye-i Sahane (Haydarpasa) Kulliyesi, Selimiye Mah. Tibbiye Cad. No: 38, 34668 Uskudar, Istanbul, Turkey

**Keywords:** Conjunctival swab, COVID-19, Nasopharyngeal swab, Pneumonia, SARS-CoV-2

## Abstract

**Background:**

The aim of this study was to demonstrate the presence of the virus in tear and conjunctival secretions of clinically-confirmed COVID-19 pneumonia patients.

**Methods:**

This prospective study was conducted at Bakirkoy Dr. Sadi Konuk Training and Research Hospital (2020/190). Nasopharyngeal and ocular samples were obtained by swab technique and investigated by RT-PCR.

**Results:**

A total of 83 patients were included. The mean age was 61.88 ± 16.04 years. 28.92% of the patients had mild, 65.06% moderate and 6.02% severe pneumonia radiologically. RT-PCR was positive in 31 (37.35%) patients in the first nasopharyngeal swabs and in 19 (22.89%) in the second swabs. 17 of 19 patients had positive both first and second nasopharyngeal swabs; only the second swabs of two patients were positive. The first conjunctival swabs RT-PCR were positive in 5 out of 83 clinically-confirmed patients or 33 laboratory-confirmed patients (rates: 6.02% and 15.15%). There were no positives detected in the second conjunctival swabs.

**Conclusions:**

SARS-CoV-2 can be detected in the conjunctival swabs of patients with COVID-19 pneumonia.

## Background

When the World Health Organization’s China Office reported cases of pneumonia of unknown etiology resembling viral pneumonia in Wuhan, Hubei Province, China on December 31, 2019, no one could have predicted that one of the greatest outbreaks of the last century had begun. But shortly after its emergence, the whole world would realize what a great threat it faced [1, 2]. On January 7, 2020, the potential causative agent was identified by Chinese authorities as a novel coronavirus (2019-nCoV) that had not been previously identified in humans [2]. On January 30, 2020, the World Health Organization (WHO) declared the outbreak to be a public health emergency of international concern. On February 11, 2020, the Coronaviridae Study Group of the International Committee on Taxonomy of Viruses named the etiologic agent of COVID-19 as “severe acute respiratory syndrome-related coronavirus 2 (SARS-CoV-2) because of its close similarity to SARS-CoV. On the same day, the WHO officially named the infection as the disease COVID-19, shortened form of “coronavirus disease 2019. On March 11, 2020, WHO announced the COVID-19 outbreak as the first pandemic caused by a coronavirus due to the alarming levels of spreading and severity in 114 countries [3]. In our country (Turkey) the first confirmed COVID-19 case was announced by the Turkish Health Minister on March 11, 2020 [4]. Despite all the global measures taken and all efforts in diagnosis and treatment, the number of people suffering from the disease and dying has been increasing day by day and the danger has been continuing. Scientists and researchers have been working with extraordinary diligence to unravel the mystery of the disease and the virus, and to prevent its known consequences since its inception.

The primary transmission of COVID-19 is from human-to-human through respiratory droplets and contact routes. Other possible routes of transmission are not clearly known and controversial [5, 6]. Anatomically, the eye is an organ that is easily exposed to respiratory viruses directly or indirectly and also connected to the upper respiratory tract through the nasolacrimal canal (NLC). Transmission of SARS-CoV-2 through infected ocular tissue or secretions has been a controversy, but it is supposed that the nasolacrimal system can work as a natural canal for viruses to pass from the eye to the upper respiratory tract or vice versa. Thus, ocular tissue and secretions may be a potential source of SARS-CoV-2 [7, 8].

This study aimed to investigate the presence of SARS-CoV-2 RNA in tear and conjunctival secretions of patients with clinically-confirmed COVID-19 pneumonia by using quantitative real time reverse transcription-polymerase chain reaction (RT-PCR) test.

## Materials and methods

This prospective observational case series study was conducted in 83 hospitalized patients with clinically-confirmed COVID-19 pneumonia at Bakirkoy Dr. Sadi Konuk Training and Research Hospital between May 10 and July 15, 2020, in Istanbul, Turkey.

The inclusion criteria were as follows: (1) Patients older than 18 years of age, (2) patients confirmed as COVID-19 pneumonia following radiological and clinical evaluation and decided to be hospitalized, (3) patients who did not receive any treatment for COVID-19, (4) patients whose nasopharyngeal samples and, tear and conjunctival secretion samples were obtained at the same time (within 3 h) for RT-PCR tests. The exclusion criteria were: (1) outpatients with pneumonia, (2) intensive care unit patients, (3) COVID-19 patients without pneumonia, (4) patients ≤ 18 years of age. Pneumonia was classified radiologically as mild (< 25% involvement), moderate (26–74% involvement) and severe (> 75% involvement) by experienced radiologists according to the total severity scores of the lung involvement on CT using a semi-quantitative scoring system based on international standards [9].

Nasopharyngeal and ocular secretion samples of all patients were collected on the first day of hospitalization and on the second (considering the discharge status) or the third day. Body temperatures of all patients were measured before conjunctival swab procedures. Nasopharyngeal samples were taken by the experienced healthcare workers, and ocular samples were taken by a senior ophthalmologist (YK) wearing personal protective equipment. Tear and conjunctival secretions were collected with conjunctival swab technique. Without topical anesthesia, the lower lid was pulled down slightly, and a commercial disposable sterile cotton-tipped swab was placed in the lower fornix. Ocular samples were collected by gently moving the swab from the nasal to the temporal by rotating it around itself. The end of the swab stick was broken and placed in a viral transport tube. Samples from both eyes were taken separately and combined in the same tube. Gloves were changed during sampling to avoid cross-contamination.

Demographic, clinical, laboratory and radiologic data were obtained from patients’ electronic medical records and from records kept during the samplings.

### Quantitative real-time PCR analysis

SARS-CoV-2 RNA detection in nasopharyngeal and ocular samples was performed by quantitative real-time polymerase chain reaction (RT-PCR). All samples taken for genomic RNA isolation were transferred to Biospeedy transfer tubes (Bioeksen, Turkey) containing 2 ml of nucleic acid preservative liquid and transported to the laboratory at 2–8 °C. All specimens were processed in biosafety level-3 (BSL-3) with full personal protective equipment. QIAsymphony DSP Virus/Pathogen Kits and a QIAsymphony isolation device (Qiagen, Germany) were used for RNA extraction. All ocular samples were studied two times with Bio-Speedy® Direct RT-qPCR SARS-CoV-2 Kits (Bioeksen R&D Technologies Inc., Turkey) targeting RdRp gene in nucleic acid isolates obtained from eye samples and one time with The DirectDetect™ SARS-CoV-2 qPCR Kits, PCR-Fluorescence Probe, (Coyote Bioscience Co., Ltd; China) targeting Orf1ab gene and N gene in accordance with kit protocols. All nasopharyngeal samples (first or second) were studied with only one of both tests and only once. For Bio-Speedy® Direct RT-qPCR SARS-CoV-2, the conditions consisted of 1 cycle of 5 min at 52 °C; then 1 cycle of 10 s at 95 °C and followed by 40 cycles of 1 s at 95 °C, of 30 s at 55 °C. The DirectDetect™ SARS-CoV-2 qPCR Kit, the conditions consisted of 1 cycle of 5 min at 42 °C; following this stage 15 cycle of 10 s at 95 °C, of 15 s at 50 °C, then 1 cycle of 1 min at 95 °C; and this was followed by 30 cycles of 10 s at 95 °C, of 30 s at 55 °C. RNA amplifications were performed using the Rotor-Gene Q RT-PCR cyler (Qiagen, Germany). For SARS CoV-2 RNA detection, FAM for the RdRp gene in Bio-Speedy® Direct RT-qPCR SARS-CoV-2, FAM for the ORF1ab gene, and ROX for the N gene were used in DirectDetectTM SARS-CoV-2. IC/HEX channels for RNase P gene were used as an internal control in both kits.

*The results were interpreted according to the protocols of the kits as follows:* In Bio-Speedy® Direct RT-qPCR SARS-CoV-2; for positive control, the detection curves of FAM and HEX channels should have a significant exponential amplification curve with the Ct < 38. For negative control, the Ct value in FAM and HEX channels should be undetermined without a significant amplification curve. In The DirectDetect™ SARS-CoV-2 qPCR Kit; for positive control, the detection curves of FAM, ROX channels should have a significant exponential amplification curve with the Ct value ≤ 25. For negative control, the Ct value in FAM and ROX channels should be undetermined without a significant amplification curve. The VIC/HEX channel should have a significant exponential amplification curve with the Ct value ≤ 25. Also, according to the kit protocols, in the samples whose controls were suitable, Ct value < 38 for Bio-Speedy® Direct RT-qPCR SARS-CoV-2, Ct value ≤ 29 for The DirectDetect™ SARS-CoV-2 qPCR Kit and those with exponential amplification curve were defined as positive.

### Statistical analysis

Statistical analysis was performed using SPSS Statistics for Windows, Version 25.0 (IBM Corp., Armonk, New York, USA). The Kolmogorov–Smirnov test was used in order to assess normality (if *p* > 0.05, the data were normally distributed). Continuous variables were expressed as mean ± standard deviation (SD), median and ranges. Categorical variables were summarized as counts and percentages. Differences in means were compared using the independent paired t test. The *p* < *0.05* was considered significant.

## Results

A total of 83 patients (38 females and 45 males) were included in the study. Details of patient characteristics of this study are presented in Table 1. The mean ± SD of age was 61.88 ± 16.04 (range 22–89) years. There was no statistical difference between males and females in terms of mean age distribution (*p* = *0.248*). Only 16 patients (19.3%) had an exposure history of close contact with infected COVID-19 cases. The median admission period of the patients was 3.00 (range 0–10) days. The most common complaint was cough that was observed in 42.17% of patients. The other three most common complaints were dyspnea in 36.15%, fever in 32.53%, and fatigue in 27.71%. Only 1 (1.21%) patient had no symptoms. The most common co-existing diseases were heart diseases (42.17%), hypertension (33.74%), and diabetes mellitus (26.50%). Twenty-one patients had no comorbidity. For the severity assessment of COVID-19 pneumonia radiologically, 28.92% of patients were classified as mild, 65.06% as moderate, and 6.02% as severe pneumonia. Lung involvement was bilateral in 92.77% of them. Demographic data of the patients were shown in Table 2.

**Table 1:** Summary of the clinically-confirmed COVID-19 pneumonia patients' characteristics

Case	Age	Sex	NP RT-PCR (1)	Eye RT-PCR (1)	NP RT-PCR (2)	Eye RT-PCR (2)	Interval	Temperature (1)	Symptoms	Days S-A	History of contacts	Coexisting disorder (chronic medical illness)	Treatments	Temperature (2)	Chest CT lung involvement	Disease severity status
1	45	F	***Positive***	Negative	Negative	Negative	3	36.4	Dyspnea, hypotension	7	Yes	Focal segmental glomerulosclerosis	HQ	36.2	Bilateral	Moderate
2	22	M	Negative	Negative	Negative	Negative	3	37.3	Cough, dyspnea, fever, headache	7	No	Asthma, migraine	HQ	36.4	Bilateral	Mild
3	66	M	***Positive***	Negative	***Positive***	Negative	3	38	Fever, fatigue	4	Yes	Diabetes mellitus, hyperlipidemia	HQ + Favipiravir	36.7	Bilateral	Moderate
4	61	F	Negative	Negative	Negative	Negative	3	36.7	Confusion, dispne	1	Yes	Diabetes mellitus, hypertension, hyperlipidemia, myocardial infarction	HQ + Favipiravir	36.6	Bilateral	Moderate
5	49	M	Negative	Negative	Negative	Negative	2	36	Abdominal pain, vomiting, poor appetite	5	No	Colorectal cancer (liver metastasis, cemoteraphy)	HQ + Antibiotic	36	Bilateral	Moderate
6	84	M	Negative	Negative	**Positive**	Negative	2	36.7	Chills, vomiting, dyspnea	1	No	No comorbidity	HQ + Antibiotic + Favipiravir	36.6	Bilateral	Severe
7	54	F	Negative	Negative	Negative	Negative	2	36.7	Cough, dyspnea	2	No	Diabetes mellitus, hypertension, chronic kidney failure, heart arrhythmia	HQ + Antibiotic + Favipiravir	36.7	Bilateral	Moderate
8	88	F	***Positive***	***Positive***	***Positive***	**Negative**	2	36.3	Asymptomatic (positive case detection at home)	0	Yes	Hypertension, coronary artery disease, chronic kidney failure	HQ	36.2	Bilateral	Mild
9	76	F	***Positive***	***Positive***	***Positive***	**Negative**	2	36.5	Fever, cough, fatigue, dry of throat	5	No	Diabetes mellitus, hypertension	HQ + Antibiotic	36.7	Bilateral	Moderate
10	48	M	Negative	Negative	Negative	Negative	2	36	Speech pelteking, syncope, urinary incontinence	1	No	Cerebrovascular diseases, bladder tumor (newly diagnosed)	HQ + Antibiotic	36.5	Bilateral	Moderate
11	83	F	Negative	Negative	Negative	Negative	2	36.5	Dyspnea, fatigue, foot swelling	5	No	Hypertension, congestive heart failure, coronary artery disease, gut syndrome	HQ + Antibiotic + Favipiravir	36.4	Bilateral	Severe
12	32	F	Negative	Negative	Negative	Negative	2	36	Dyspnea, fatigue, poor appetite, generalized myalgia	7	No	No comorbidity	HQ + Antibiotic	36.5	Bilateral	Moderate
13	52	F	Negative	Negative	Negative	Negative	3	37.5	Chills	1	No	Lung cancer (cemoteraphy)	HQ + Antibiotic	36	Bilateral	Moderate
14	87	M	Negative	Negative	Negative	Negative	3	36.7	Confusion, loss of power and strength in the extremities	0	No	Hypertension, chronic kidney failure, congestive heart failure, heart arrhythmia	HQ + Antibiotic	36	Bilateral	Moderate
15	55	F	***Positive***	Negative	***Positive***	Negative	3	36.8	Dyspnea, fever, dry cough	3	No	Diabetes mellitus	HQ + Antibiotic	36.7	Bilateral	Moderate
16	56	M	***Positive***	Negative	***Positive***	Negative	3	38	Cough, fever	4	No	No comorbidity	HQ + Antibiotic + Favipiravir	38.3	Bilateral	Moderate
17	35	M	***Positive***	***Positive***	***Positive***	**Negative**	3	38	Headache, nausea, fever, poor appetite	3	No	No comorbidity	HQ	36.4	Bilateral	Moderate
18	56	M	Negative	Negative	Negative	Negative	3	37.6	Headache, nausea, cough, flank pain, dysuria	4	No	No comorbidity	HQ + Antibiotic	37.3	Unilateral	Moderate
19	70	M	Negative	Negative	Negative	Negative	3	36.6	Cough, back pain	5	Yes	Diabetes mellitus	HQ + Antibiotic	36.2	Bilateral	Moderate
20	31	M	Negative	Negative	Negative	Negative	3	37	Cough, dyspnea, headache, fatigue	3	No	No comorbidity	HQ + Antibiotic	36.2	Bilateral	Mild
21	63	M	Negative	Negative	Negative	Negative	3	36.8	Headache, fever, nausea, abdominal pain	1	No	Coronary artery disease, hypertension	HQ + Antibiotic	36.7	Bilateral	Moderate
22	59	F	Negative	Negative	Negative	Negative	3	36.5	Diarrhea, abdominal pain, nausea, vomiting, fever	3	No	Hypertension, hypothyroidism	HQ	36.7	Bilateral	Moderate
23	87	F	Negative	Negative	Negative	Negative	3	36.5	Dyspnea	3	No	Hypertension	HQ	36.5	Bilateral	Moderate
24	43	F	***Positive***	Negative	Negative	Negative	3	36.5	Cough, nausea, generalized myalgia, palpitation	4	Yes	Hypertension	HQ + Antibiotic	36.4	Bilateral	Moderate
25	58	M	*Positive*	Negative	***Positive***	Negative	3	36.7	Nausea, fatigue	7	Yes	No comorbidity	HQ + Antibiotic	36	Bilateral	Moderate
26	74	M	***Positive***	***Positive***	***Positive***	**Negative**	3	36.5	Fatigue, confusion poor appetite, self-talk disorder	4	No	Diabetes mellitus, hypertension	HQ + Antibiotic	37.3	Bilateral	Moderate
27	58	F	***Positive***	Negative	Negative	Negative	3	36.9	Cough, fatigue, back pain	7	Yes	Diabetes mellitus, hypertension	HQ + Antibiotic	36.6	Bilateral	Moderate
28	71	M	Negative	Negative	Negative	Negative	2	36.8	Fatigue, headache, fever	5	No	Rheumatologic disorder	HQ	36	Bilateral	Moderate
29	66	F	***Positive***	Negative	Negative	Negative	2	37	Cough, fever, nausea, fatigue	7	Yes	Hypothyroidism, heart arrhythmia	HQ + Favipiravir	36.3	Bilateral	Moderate
30	35	M	***Positive***	Negative	***Positive***	Negative	2	38.3	Fever, nausea	4	Yes	Bronchiectasis	HQ + Favipiravir	37.6	Bilateral	Moderate
31	65	M	Negative	Negative	Negative	Negative	3	37.2	Sore throat, cough	3	No	Bladder tumor, total thyroidectomy, stomach perforation surgery	HQ + Antibiotic	36.6	Bilateral	Mild
32	46	F	***Positive***	Negative	Negative	Negative	3	37.7	Fever, cough, dyspnea	7	No	Asthma	HQ	36.5	Bilateral	Moderate
33	63	M	***Positive***	Negative	***Positive***	Negative	3	37.9	Fever	5	Yes	Hypertension	HQ	37.1	Bilateral	Moderate
34	72	M	Negative	Negative	Negative	Negative	3	36.5	Fatigue, joint pain, generalized myalgia	7	No	Diabetes mellitus, hypertension, coronary artery disease	HQ	36.3	Bilateral	Mild
35	32	F	***Positive***	Negative	Negative	Negative	3	36.6	Fever, cough, headache, loss of smell	4	No	No comorbidity	HQ + Antibiotic	37.6	Bilateral	Moderate
36	72	F	Negative	Negative	Negative	Negative	3	37.5	Fever, cough	3	No	Diabetes mellitus, hypertension, asthma, coronary artery disease, hyperlipidemia	HQ	37	Bilateral	Mild
37	58	F	Negative	Negative	Negative	Negative	3	36.5	Chills, myalgia, headache	1	No	No comorbidity	HQ + Antibiotic	36.3	Bilateral	Mild
38	64	M	Negative	Negative	Negative	Negative	3	36.7	Headache, fatigue	2	No	Coronary artery disease (history of cardiac anjio)	HQ	36.4	Bilateral	Moderate
39	58	M	***Positive***	***Positive***	***Positive***	**Negative**	3	38.9	Fever	3	No	Heart valve disease (heart valve replacement surgery)	HQ	36.2	Bilateral	Mild
40	61	M	Negative	Negative	Negative	Negative	3	36.8	Cough, dizziness, dyspnea, dry of throat, diarrhea	5	No	Coronary artery disease (coronary angioplasty and stenting)	HQ + Antibiotic	36.4	Unilateral	Mild
41	83	F	***Positive***	Negative	Negative	Negative	3	37.4	Chills, fever	3	No	Coronary artery disease, asthma	HQ + Antibiotic	36.2	Bilateral	Moderate
42	79	F	Negative	Negative	Negative	Negative	3	36.5	Nausea, vomiting, cellulite on the left leg	7	No	Asthma, coronary artery disease, hypertension	HQ + Antibiotic	36	Bilateral	Moderate
43	65	F	Negative	Negative	Negative	Negative	2	36.2	Epistaxis	4	No	Splenectomy, superior mesenteric venous thrombosis	HQ + Antibiotic	36	Bilateral	Moderate
44	42	M	Negative	Negative	Negative	Negative	3	36.5	Chills, fatigue, headache	3	Yes	No comorbidity	HQ	36.3	Bilateral	Mild
45	71	M	***Positive***	Negative	***Positive***	Negative	3	38	Cough, fever	10	No	Hypertension, coronary artery disease	HQ + Antibiotic	36.5	Bilateral	Mild
46	64	M	Negative	Negative	Negative	Negative	2	36.5	Abdominal distention, dyspnea, fever, cough	4	No	No comorbidity	HQ	36.9	Bilateral	Mild
47	81	F	***Positive***	Negative	***Positive***	Negative	2	36.5	Nausea, vomiting	0	No	Diabetes mellitus	HQ	36.5	Bilateral	Mild
48	42	M	Negative	Negative	Negative	Negative	2	36.7	Fever, cough, dyspnea	1	No	No comorbidity	HQ + Antibiotic	36	Bilateral	Moderate
49	66	M	Negative	Negative	Negative	Negative	2	36.5	Cough, fatigue, vomiting	5	No	No comorbidity	HQ + Antibiotic	36.5	Bilateral	Moderate
50	89	M	Negative	Negative	Negative	Negative	2	36.5	Vomiting, diarrhea	0	No	Lung cancer, chronic obstructive pulmonary disease, Alzheimer's disease	HQ + Antibiotic	36.4	Bilateral	Moderate
51	73	F	***Positive***	Negative	***Positive***	Negative	3	36.5	Fatigue, loss of taste	7	No	Diabetes mellitus, hypertension,coronary artery disease	HQ	36	Bilateral	Mild
52	62	F	***Positive***	Negative	***Positive***	Negative	3	37.2	Fever, sore throat	2	No	No comorbidity	HQ + Antibiotic	36	Unilateral	Mild
53	86	F	***Positive***	Negative	Negative	Negative	3	36.6	Dyspnea	3	No	Congestive heart failure, coronary artery disease,hypertension, chronic kidney failure	HQ + Antibiotic + Favipiravir	36.2	Bilateral	Moderate
54	48	F	Negative	Negative	Negative	Negative	3	36.2	Chest pain, dyspnea	1	No	Pulmonary embolism	HQ + Antibiotic	36.4	Bilateral	Moderate
55	47	M	***Positive***	Negative	Negative	Negative	3	36.5	Headache, hypotension	2	No	Hypertension, hypothyroidism	HQ + Antibiotic	36.3	Bilateral	Moderate
56	76	F	Negative	Negative	Negative	Negative	3	36.6	Dyspnea, fever, abdominal pain	1	No	Coronary artery disease	HQ	36.3	Bilateral	Mild
57	70	M	Negative	Negative	Negative	Negative	3	36	Chest pain	0	No	Diabetes mellitus, coronary artery disease, chronic kidney failure	HQ	36.7	Bilateral	Mild
58	77	M	Negative	Negative	Negative	Negative	3	39.8	Fever, dyspnea, vomiting	1	No	Coronary artery disease (heart bypass surgery)	HQ + Antibiotic + Favipiravir	36.1	Bilateral	Severe
59	65	F	Negative	Negative	Negative	Negative	3	36.5	Cough, chills	4	No	No comorbidity	HQ + Antibiotic	36.6	Bilateral	Mild
60	59	M	Negative	Negative	Negative	Negative	2	36	Dyspnea	2	No	Coronary artery disease	HQ + Antibiotic	36	Bilateral	Moderate
61	73	M	Negative	Negative	Negative	Negative	3	36.9	Cough, dyspnea, legs swelling, chest pain	2	No	Hypertension, coronary artery disease (heart bypass surgery)	HQ	37.1	Bilateral	Moderate
62	43	M	Negative	Negative	Negative	Negative	3	37.1	Fever, headache, diarrhea, nausea, dry of throat	2	Yes	No comorbidity	HQ	36	Bilateral	Moderate
63	50	F	Negative	Negative	Negative	Negative	3	37.3	Fatigue, cough, fever, dyspnea	5	No	No comorbidity	HQ + Antibiotic + Favipiravir	36.3	Bilateral	Severe
64	59	M	Negative	Negative	Negative	Negative	3	36	Dyspnea	3	No	Distal Pankreatektomi ve splenektomi (pancreatic cancer?)	HQ	36.6	Bilateral	Moderate
65	78	F	Negative	Negative	Negative	Negative	3	36.2	Abdominal pain	10	No	Coronary artery disease	HQ + Antibiotic	36	Bilateral	Mild
66	86	F	Negative	Negative	Negative	Negative	3	36.4	Dyspnea	1	No	Heart failure, coronary artery disease, myocardial infarction	HQ + Favipiravir	36	Bilateral	Moderate
67	71	M	***Positive***	Negative	***Positive***	Negative	3	36.3	Cough, dyspnea	7	Yes	Hypertension, coronary artery disease	HQ + Favipiravir	36.3	Unilateral	Moderate
68	71	F	***Positive***	Negative	Negative	Negative	3	36.5	Cough, fatigue, headache	7	Yes	Goitre	HQ + Antibiotic	36.3	Bilateral	Mild
69	83	F	Negative	Negative	Negative	Negative	3	36	Cough expectorate, fatigue	10	No	Diabetes mellitus, hypertension, coronary artery disease, chronic bronchitis	HQ + Antibiotic	36.8	Bilateral	Moderate
70	30	F	Negative	Negative	***Positive***	Negative	3	37.5	Cough, fatigue	7	No	No comorbidity	HQ + Antibiotic	36.2	Bilateral	Moderate
71	77	M	Negative	Negative	Negative	Negative	3	36.5	Dizziness, weakness of the right side	0	No	Diabetes mellitus, cerebrovascular diseases	HQ	36.3	Unilateral	Mild
72	70	F	Negative	Negative	Negative	Negative	3	36	Dyspnea, cough	1	No	Coronary artery disease, hypertension, asthma, diabetes mellitus	HQ + Antibiotic	35.5	Bilateral	Moderate
73	86	M	Negative	Negative	Negative	Negative	3	37.3	fever, chills, dyspnea	3	No	Coronary artery disease, hypertension, diabetes mellitus, chronic kidney failure	HQ + Antibiotic	36.7	Bilateral	Moderate
74	61	M	Negative	Negative	Negative	Negative	3	36	Chills, generalized myalgia, cough	1	No	No comorbidity	HQ + Antibiotic	36	Bilateral	Moderate
75	56	F	Negative	Negative	Negative	Negative	3	36	Fatigue, myalgia, back pain	3	No	Coronary artery disease, hypertension	HQ	36.3	Bilateral	Mild
76	65	M	Negative	Negative	Negative	Negative	3	36.1	Sudden shortness of breath, disartria, dizziness, sweating	0	No	Diabetes mellitus, hypertension	HQ	36.4	Bilateral	Moderate
77	52	M	***Positive***	Negative	***Positive***	Negative	3	36.1	Dry cough	3	No	Diabetes mellitus	HQ + Antibiotic + Favipiravir	36.4	Bilateral	Moderate
78	64	F	***Positive***	Negative	Negative	Negative	3	36.6	cough, dyspnea, fatigue	7	No	Diabetes mellitus, hypertension	HQ	36.5	Bilateral	Moderate
79	70	M	Negative	Negative	Negative	Negative	3	36.6	Cough, hemoptysis	0	No	Coronary artery disease, cerebrovascular diseases	HQ	36.5	Bilateral	Mild
80	63	F	***Positive***	Negative	Negative	Negative	3	36.5	Fatigue, palpitation	4	No	Diabetes mellitus	HQ + Favipiravir	36.2	Bilateral	Severe
81	46	M	***Positive***	Negative	Negative	Negative	3	36.3	Cough, dyspnea, fatigue, sweating	7	Yes	No comorbidity	HQ	36.6	Bilateral	Moderate
82	55	M	***Positive***	Negative	Negative	Negative	3	36.2	Cough, dyspnea, chills	4	No	Cerebrovascular diseases, diabetes mellitus, coronary artery disease (bypass surgery)	HQ + Antibiotic	36	Bilateral	Mild
83	27	M	Negative	Negative	Negative	Negative	3	36.8	Cough, fever	3	No	No comorbidity	HQ + Antibiotic	36.6	Unilateral	Moderate

*NP* nasopharyngeal, *Eye*: tear and conjunctival secretions, *RT-PCR* reverse transcription polymerase chain reaction, *Days S-A* days from onset of symptoms to admission

**Table 2:** Demographic and clinical characteristics of the clinically-confirmed COVID-19 pneumonia patients

Characteristics	All patients (*n* = 83)	
	Mean (SD)	Range
**Age, years**		
All	61.88 (16.04)	22–89
Female	64.11 (16.17)	30–88
Male	60 (15.86)	22–89
**Temperature at the sampling** (Celcius, °C)		
Temperature (1)	36.78 (0.69)	36.00–39.80
Temperature (2)	36.44 (0.43)	35.50–38.50
**Days from onset of symptoms to admission (Days S-A)**	3.65 (2.54)	0–10

	Patients (*n*)	Percentage (*%)*
**Sex**		
Female	38	45.78
Male	45	54.22
**History of contacts**		
Yes	16	19.28
No	67	80.72
**Symptoms**		
Asymptomatic	1	1.205
Cough	35	42.17
Dyspnea	30	36.15
Fever	27	32.53
Fatigue	23	27.71
Headache	13	15.66
Nausea	11	13.25
Vomiting	9	10.83
Chills	8	9.64
Myalgia or arthralgia	7	8.43
Sore or dry throat	6	7.23
Abdominal pain or distention	6	7.23
Poor appetite	4	4.82
Diarrhea	4	4.82
Confusion	3	3.61
Back pain	3	3.61
Dizziness	3	3.61
Chest pain	3	3.61
Sweating	2	2.41
Hypotension	2	2.41
Palpitation	2	2.41
Neurological symptoms (speech pelteking, disartria, loss of power and strength in the extremities, syncope, weakness of the right side, self-talk disorder)	6	7.23
Other symptoms (cellulite on the leg, foot swelling, legs swelling, urinary incontinence, flank pain, dysuria, loss of smell and taste, hemoptysis, epistaxis)	10	12.05
Chronic medical illness		
Hypertension	28	33.74
Diabetes mellitus	22	26.5
Coronary artery disease	25	30.12
Other heart diseases	10	12.05
Chronic kidney disease	7	8.43
Asthma	6	7.23
Other respiratory system disease	6	7.23
Thyroid diseases	5	6.02
Cerebrovascular diseases	4	4.82
Malignancy (except lung CA)	4	4.82
Hyperlipidemia	3	3.61
Rheumatologic disorder	1	1.205
Autoimmune disorder-Gut	1	1.205
Others (splenectomy, stomach surgery)	2	2.41
No comorbidity	21	25.3
**Lung involvement-chest CT**		
Unilateral	6	7.23
Bilateral	77	92.77
**Disease severity status—radiologically**		
Mild	24	28.92
Moderate	54	65.06
Severe	5	6.02
**Treatments**		
Hydroxychloroquine	28	33.74
Hydroxychloroquine + Antibiotic	40	48.19
Hydroxychloroquine + Antibiotic + Favipiravir	8	9.64
Hydroxychloroquine + Favipiravir	7	8.43

SARS-CoV-2 RNA was detected in the first nasopharyngeal swabs in 31 of 83 (37.35%) patients. Other patients were confirmed as COVID-19 patients based on their clinical and radiological findings. For the second nasopharyngeal swab samples, 19 (22.89%) patients were RT-PCR positive (Table 3). The first nasopharyngeal swabs were positive in 17 of 19 patients except for 2 (Case 6 and 70; Table 1). Thus, the number of laboratory-confirmed patients was 33 [31(first) + 2 (second)]. RT-PCR was positive only in 5 (6.02%) out of 83 patients for first conjunctival swabs samples (Table 3). RT-PCR tests of the first nasopharyngeal swabs obtained from these 5 patients were positive. The rate of patients with conjunctival swabs in positive nasopharyngeal group for the first swabs was 16.13%. However, when the other two positive patients in the second nasopharyngeal swabs were added, the number of patients with laboratory-confirmed COVID-19 pneumonia was 33, therefore the rate became 15.15%. The virus RNA could not be detected in any of the second conjunctival swab samples. RT-PCR test Ct values of 5 cases with SARS-CoV-2 RNA detected in their first tear and conjunctival secretion swabs were shown in Table 4.

**Table 3:** Nasopharyngeal and ocular secretions RT-PCR test results in clinically-confirmed COVID-19 pneumonia patients

Test	Positive	Negative
Fist nasopharyngeal RT-PCR	31 (37.35%)	52 (62.65%)
*First tear and conjunctival secretions RT-PCR*	*5 (6.02%)*	*78 (93.98%)*
Second nasopharyngeal RT-PCR	19 (22.89%)	64 (77.11%)
*Second tear and conjunctival secretions RT-PCR*	*0 (0%)*	*83 (100%)*

ocular tests have been written in italics to increase recognizability

*RT-PCR* reverse transcription polymerase chain reaction

**Table 4:** Cycle threshold (Ct) values in RT-PCR tests of patients with SARS-CoV-2 RNA detected in their first conjunctival and nasopharyngeal swabs

Manufacturer	Version	Case	Eye RT-PCR (1)			PC (FAM)	PC (HEX)	NC	NP RT-PCR (1)	
			Green (FAM)	Yellow (HEX)	Orange (ROX)				Green (FAM)	Yellow (HEX)
Bioeksen, Turkey	V-4	Case 8	28.59	25.86		20.91	22.70	NEG (NTC)	15.71	12.01
Coyote, China	V-1		21.80	18.22	18.02	16.73	24.20	NEG (NTC)		
Bioeksen, Turkey	V-2	Case 9	37.95	25.03		29.80	28.07	NEG (NTC)	17.82	10.85
Coyote, China	V-1		24.65	15.47	20.10	16.73	24.20	NEG (NTC)		
Bioeksen, Turkey	V-2	Case 17	32.85	26.35		29.80	28.07	NEG (NTC)	27.45	10.59
Coyote, China	V-1		27.30	17.86	20.32	16.73	24.20	NEG (NTC)		
Bioeksen, Turkey	V-4	Case 26	28.06	23.87		20.91	22.70	NEG (NTC)	19.38	21.92
Coyote, China	V-1		20.30	14.43	16.53	16.73	24.20	NEG (NTC)		
Bioeksen, Turkey	V-4	Case 39	28.66	24.65		20.91	22.70	NEG (NTC)	13.84	20.56
Coyote, China	V-1		20.89	17.44	16.77	16.73	24.20	NEG (NTC)		

None of the patients in this study had any ocular symptoms or findings.

## Discussion

It is well known that SARS-CoV-2 virus is primarily transmitted between people through respiratory droplets and contact routes [6]. Droplets can be formed through coughing, sneezing, singing, breathing, and speaking. Nevertheless, possible routes of transmission such as airborne, fecal–oral, vertical, sexual, ocular are still under discussion [5, 6, 10].

The potential for the ocular route of transmission and the presence of the SARS-CoV-2 in ocular tissues has been recently investigated. Transfer of the virus to ocular tissues can take place mainly in four ways: (1) by direct exposure to virus containing infectious droplets, (2) by contaminated hands or fomites, (3) by spreading due to viremia to the lacrimal gland and other eye tissues with dense vascularity (controversial), (4) by spreading from the upper respiratory tract to the lacrimal sac via the NLC and from there to the eye (controversial) [7]. NLC, a functional natural canal that connects the eye and respiratory system, is the main route of transmission from the eye to the respiratory system [7, 11]. In an experimental animal study published recently, it has been shown that the virus can replicate in conjunctival cells and cause viral pneumonia through the eye [12]. In addition to all these, it should be emphasized that the eye has some features mentioned below that limits its being an alternative route of transmission and reservoir organ. Firstly, the tear is constantly renewed, lost by evaporation, is continuously drained by the NLC, and its volume is also very low. Secondly, ocular secretions have a strong local immune system (lactoferrin, immunoglobulins, sialic acid and etc.) against microorganisms (including coronavirus). Thirdly, SARS like coronavirus is known to be a member of the genus *Betacoronavirus* and subgenus *Sarbecovirus* and is an enveloped positive-sense single-stranded RNA virus that enters its host cell by binding to the ACE-2 receptor. These receptors have been shown to be present in small amounts (at least 50% lower than other tissues) in conjunctival mucosa cells, and also their ability to bind to the virus is very poor [7, 12–14]. Moreover, the virus load is thought to be found in ocular secretions much less than in throat secretions and if the virus is present in ocular secretions, it is not known how long the virus may also persist [7, 12–15]. Factors such as technical errors and difficulties in sampling, handling, processing, and evaluation, the stage of the disease at the time the samples are taken, and the specificity and sensitivity of the tests may also negatively affect the test results and cause lower positivity rates [7, 14].

During the pandemic, the first case of conjunctivitis related to COVID-19 was reported by a Chinese respiratory specialist who visited Wuhan as a member of the national expert panel on pneumonia. He wore an N95 mask, but did not wear anything to protect his eyes while working with his patients. His first clinical sign of COVID-19 pneumonia was unilateral conjunctivitis. He claimed that SARS-CoV-2 probably first infected the conjunctiva, then spread through ocular secretions and caused viral pneumonia [7, 16]. In a study by the China Medical Treatment Expert Group, conjunctival congestion was reported in 9 (0.8%) out of 1099 patients with laboratory-confirmed COVID-19 from 552 different hospitals [17]. It should be underlined that the diagnosis of conjunctivitis was not based on examinations performed by ophthalmologists in that study. In a study by ophthalmologists in China, conjunctival congestion was reported in 27 (5.0%) out of 535 confirmed COVID-19 patients. The initial symptom of 4 patients was conjunctival congestion. SARS-CoV-2 was detected in nasopharyngeal swabs in 18 (66.7%) out of 27 patients and 343 (64.0%) out of 535 patients [18]. On the other hand, there are some studies showing the presence of virus in conjunctival swab samples by RT-PCR test. Xia et al. [19] detected the SARS-CoV-2 by RT-PCR in two conjunctival swab samples collected from only one patient with conjunctivitis among 30 laboratory-confirmed COVID-19 pneumonia patients. Fifty-eight samples obtained from other patients were all negative. Nasopharyngeal and ocular samples were taken at the same time on the first day and on the 2nd or 3rd day. Fang et al. [20] collected nasal, blood, stool, urine, saliva, and tear samples from 32 laboratory-confirmed COVID-19 patients and detected the presence of SARS-CoV-2 by RT-PCR in conjunctival samples of 5 patients (15.63%). Liang and Wu et al. [21] evaluated conjunctival swabs from 37 confirmed COVID-19 patients, three of whom had conjunctivitis. Only one conjunctival swab (2.70%) from a patient with severe pneumonia (according to the Chinese COVID-19 diagnostic protocol) without conjunctivitis yielded a positive result. In a study conducted by Wu et al. [22] 12 out of 38 (31.58%) clinically-confirmed COVID-19 patients had ocular findings consistent with conjunctivitis, and two patients (5.26%) with conjunctivitis were positive for SARS-CoV-2 in conjunctival swabs as well as nasopharyngeal swabs. Karimi et al. [23] detected the presence of the virus in nasopharyngeal samples of 30 (69.8%) and in tear samples of 3 (7%) patients out of 43 clinically-confirmed COVID-19 patients with pneumonia. Nasopharyngeal RT-PCRs of those three patients were also positive. One patient with bilateral conjunctivitis had a negative conjunctival swab. Kumar et al. [24] reported that only one (2.23%) of the conjunctival swabs taken from 45 patients with COVID-19 had detectable SARS CoV-2 levels by RT PCR. In a preprint posted at MedRxiv by Zhou et al. [25], conjunctival swab sample of 1 patient was found positive, and conjunctival swab samples of 2 patients probable positive (suspicious positive) in 67 confirmed or suspected cases of COVID-19 pneumonia (mostly healthy workers). None of the three patients had ocular symptoms. One patient whose first symptom was conjunctivitis had a negative conjunctival swab. In another preprint posted at MedRxiv, Sun et al. [26] evaluated 72 laboratory-confirmed COVID-19 pneumonia patients and reported that SARS-CoV-2 was detected in a conjunctival sample of only one patient. This patient was an emergency department nurse and bilateral acute conjunctivitis was her first symptom of COVID-19 pneumonia. In addition, a small number of "Case Report" studies published including COVID-19 associated conjunctivitis cases detected SARS-CoV-2 by RT-PCR in conjunctival swab samples [27–29]. In one of those studies by Colavita et al. [28], it was reported that the viable virus was isolated from conjunctival swab samples and exhibited a cytopathic effect in Vero E6 cells.

A few studies containing results contrary to the studies mentioned above have also been reported. In a study conducted in Singapore, a total of 64 samples were obtained from 17 laboratory-confirmed COVID-19 patients from day 3 to day 20 following the first symptoms of the disease, and none of these samples gave positive results by viral isolation and RT-PCR [8]. In another study conducted by Deng et al. [15] in China, no SARS-CoV-2 could be detected by RT-PCR in conjunctival swab samples from 114 patients with clinically-confirmed COVID-19 pneumonia.

In the present study, 83 hospitalized patients with clinically confirmed COVID-19 pneumonia were evaluated to detect the presence of SARS-CoV-2 in tears and conjunctival secretions. The mean age of the patients was 61.88 ± 16.04 years, and approximately 75% of them suffered from co-existing chronic diseases. Radiologically, the majority of patients had mild to moderate pneumonia and 92.77% of them had bilateral lung involvement. Although conjunctivitis was not an exclusion criterion, no conjunctivitis was detected in any patients. As in previous studies, the most common complaints of the patients were cough, fever, dyspnea and fatigue respectively. In Tables 1 and 2, it is seen that the body temperatures of the patients measured at the time of sampling are not high because of using antipyretic and other medications. Regardless of whether they were laboratory-confirmed or not, all patients were treated according to the “COVID-19 pneumonia management guide” prepared by the Ministry of Health's Science Committee [4].

In this study, tear and conjunctival samples and nasopharyngeal samples were obtained simultaneously for RT-PCR on the admission and on the 2nd or the 3rd day after hospitalization. In total, 166 tear and conjunctival samples and 166 nasopharyngeal samples were investigated. For first nasopharyngeal samples, the presence of the SARS-CoV-2 by RT-PCR was detected in 31 out of 83 patients (37.35%). However, SARS-CoV-2 RNA was detected in the conjunctival swabs of only 5 of the 83 patients with a rate of 6.02%. All 5 cases (Case 8, 9, 17, 26 and 39; Table 1) who were positive for the first tear and conjunctival RT-PCR, were positive for the first nasopharyngeal RT-PCR as well. When considering patients with first positive nasopharyngeal RT-PCR, the conjunctival swab positivity was 5/31 (16.13%). Of the 31 patients whose first nasopharyngeal swabs were positive, only 17 were also positive in the second. Following the addition of two patients whose first nasopharyngeal RT-PCR was negative but the second was positive, the number of laboratory-confirmed patients became 33, the conjunctival swab RT-PCR positivity 15.15% in patients with laboratory-confirmed COVID-19 pneumonia. In this study, the conjunctival swab RT-PCR positivity in patients with laboratory-confirmed COVID-19 pneumonia was slightly higher than those of the studies mentioned above. One of the important results of the study was that no conjunctival swab positivity was detected from any patients whose first and second nasopharyngeal samples were negative. Another notable result was that there was a finding supporting the view that the virus could not remain in the eye secretions for a long time. All 5 patients with positive first conjunctival and nasopharyngeal swabs had positive second nasopharyngeal swabs, but had no positive second conjunctival swabs. Furthermore, when the Ct values given in Table 4 are evaluated, it is seen that the conjunctival swab Ct values are higher than the nasopharyngeal swab values. Accordingly, it can be concluded that the viral load of the nasopharynx is probably higher than those of ocular secretions.

In summary, 5 patients having conjunctival swab positive had bilateral lung involvement (Case 8 and 39 were mild, the others were moderate pneumonic), only one (Case 8) had a history of exposure close contact, and, except for one, the others had a chronic disease. Three patients underwent only hydroxychloroquine (HQ) and the other two underwent HQ + antibiotics for COVID-19 pneumonia.

The limitations of our study were the relatively small number of laboratory-confirmed COVID-19 pneumonia patients and intensive care patients who were likely to have a higher viral load were excluded from the study.

## Conclusions

This study showed that SARS-CoV-2 can be detected in conjunctival swabs of patients with clinically or laboratory confirmed COVID-19 pneumonia. The positivity of SARS-CoV-2 (15.15%) in tear and conjunctival secretions of patients with laboratory-confirmed COVID-19 pneumonia was found to be slightly higher than those of the previous studies. Also, the positivity (6.02%) in tear and conjunctival secretions of patients with clinically-confirmed COVID-19 pneumonia was found to be similar to or even slightly higher than those of the previously conducted studies. Although this is not an experimental study, when the positivity-negativity rates and Ct values are evaluated together, it can be thought that the viral load in nasopharyngeal secretions is probably higher than the ocular secretions and the virus may not remain in the ocular secretions for a long time.

## Data Availability

All data generated or analyzed during this study was included in this published article.
